# Multiple Routes of Bluetongue Virus Egress

**DOI:** 10.3390/microorganisms8070965

**Published:** 2020-06-27

**Authors:** Thomas Labadie, Edward Sullivan, Polly Roy

**Affiliations:** Department of Pathogen Molecular Biology, Faculty of Infectious and Tropical Diseases, London School of Hygiene and Tropical Medicine, Keppel Street, WC1E 7HT, London WC1E 7HT, UK; Thomas.Labadie@lshtm.ac.uk (T.L.); Edward.Sullivan@lshtm.ac.uk (E.S.)

**Keywords:** bluetongue virus, virus egress, non-structural protein, extracellular vesicles, arthropod-borne virus

## Abstract

Bluetongue virus (BTV) is an arthropod-borne virus infecting livestock. Its frequent emergence in Europe and North America had caused significant agricultural and economic loss. BTV is also of scientific interest as a model to understand the mechanisms underlying non-enveloped virus release from mammalian and insect cells. The BTV particle, which is formed of a complex double-layered capsid, was first considered as a lytic virus that needs to lyse the infected cells for cell to cell transmission. In the last decade, however, a more in-depth focus on the role of the non-structural proteins has led to several examples where BTV particles are also released through different budding mechanisms at the plasma membrane. It is now clear that the non-structural protein NS3 is the main driver of BTV release, via different interactions with both viral and cellular proteins of the cell sorting and exocytosis pathway. In this review, we discuss the most recent advances in the molecular biology of BTV egress and compare the mechanisms that lead to lytic or non-lytic BTV release.

## 1. Introduction

Bluetongue virus (BTV) is an arthropod-borne virus vectored by biting midges from the *Culicoides* genus. BTV infects both domestic and wild ruminants, and the infection induces different variations of pathogenicity, from asymptomatic infection to severe symptoms such as ecchymosis, cardiac lesions, and hemorrhages, especially in sheep. Since the 1950s, BTV has spread globally from Africa to Europe, Asia, North and South America. Further, periodic outbreaks of new serotypes cause high morbidity in livestock, often with significant mortality and consequently associate with substantial economic losses in the agricultural industry.

BTV belongs to the *Reoviridae* family, which are characterised by a non-enveloped icosahedral capsid. The ten segments of the BTV double-stranded RNA genome encode seven structural proteins (VP1, VP2, VP3, VP4, VP5, VP6 and VP7) and six non-structural proteins (NS1, NS2, NS3, NS3A, NS4 and NS5) [1,2]. During infection, VP2 and VP5, which form the outer capsid, are responsible for BTV attachment and entry into cells via the endocytic pathway. Within early and late endosome, pH-induced conformational changes of VP2 and VP5 facilitate membrane penetration of the core [3], which is then released into the cytoplasm. The core is composed of an inner capsid (comprised of VP3 and VP7), containing the transcription complexes that are constituted by VP1 (RNA-dependent RNA polymerase), VP4 (capping enzyme), VP6 (helicase/RNA packaging) and the dsRNA segments [4,5]. The non-structural proteins fulfil essential functions within the virus life cycle, but do not form part of the virion. NS1 is necessary for BTV replication and selectively enhances viral protein synthesis [6]. It also undergoes polymerisation producing large tubules in infected cells, whose functional consequence is unknown [7]. NS2 is responsible, and sufficient, for the generation of viral inclusion bodies (VIBS), large globular structures in cytoplasm of infected cells. NS2 acts as a scaffold in the cytoplasm, recruiting positive sense ssRNA transcripts, the transcription complex components, and inner capsid proteins into VIBs. VIBs are, therefore, the sites of virus replication and are believed to be the site of viral core assembly, as inner capsid protein VP3 and VP7 have been found inside VIBs [8]. The outer capsid protein VP5 is also observed in the VIBs, suggesting that the first layer of the outer capsid could be assembled in the VIBs [9,10]. In contrast, the assembly of the VP2 protein does not happen in the VIBs [11], and the localisation of core maturation and VP2 assembly remains to be determined. However, it is likely that BTV uses the cell cytoskeleton for virus particles maturation, as it was previously demonstrated that VP2 is able to interact with the vimentin intermediate filaments [12]. The NS3 protein is the only glycoprotein containing membrane domains synthesised by BTV. NS3 is believed to be synthesised at the endoplasmic reticulum (ER), and transits through the Golgi apparatus, where it becomes glycosylated, before reaching the plasma membrane [13,14]. NS3 is the major regulatory protein of virus maturation, trafficking and egress [15,16,17,18]. Here, we review the recent advances made on the routes of BTV egress and discuss the different mechanisms, and the role played by NS3.

## 2. BTV Egress: Lytic Versus Non-Lytic Release

**Figure 1:**
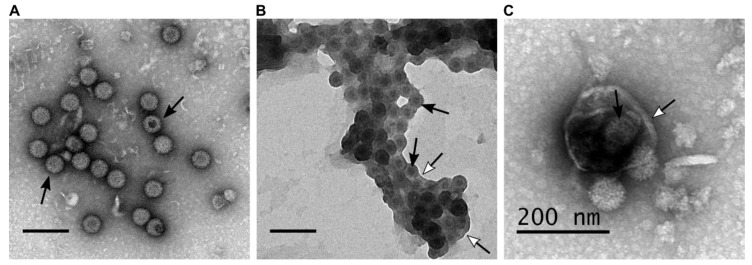
BTV particles purified from the supernatant of infected ovine PT cells (derived from domestic sheep) present different phenotypes. (**A**–**C**) Transmission electron microscopy of BTV virus particles that are (**A**) naked, (**B**) transiently associated with lipid membranes after budding at the plasma membrane, (**C**) or cloaked in EVs. Scale bars represent 200 nm, white head arrows indicate the lipid membranes, and black arrows indicate BTV particles.

During BTV infection both lytic and non-lytic virion release has been documented. Further, electron microscopy analysis revealed different forms of virion particles, naked virion particles released by cell lysis and non-lytic viruses budding through the plasma membrane [19]. In addition to individual particle budding, a recent report demonstrated virion particles are released in aggregates within extracellular vesicles (EVs), suggesting a secretory pathway [17]. These suggest that BTV is capable of different modes of virion egress which result in the different forms of virus particle (Figure 1).

Classically, non-enveloped viruses were thought to rely on lytic strategies for release, killing the infected cell in the process. In that regard, it has been shown that infection by several viruses from the *Reoviridae* family trigger cellular apoptosis [20,21,22,23,24]. In particular, BTV infection induces nuclear factor kappa B (NF-κB), caspase-3, DNA fragmentation and membrane disruption [22]. In mammalian cells, the expression of outer capsid proteins alone is sufficient to trigger apoptosis [25]. The mitogen activated protein kinase/extracellular signal-regulated kinase (MAPK/ERK) pathway, a key mediator of apoptosis, can be modulated by BTV in a strain-dependent manner [26,27]. Along with VP2, NS3 is a major determinant of BTV virulence [28]. Recently, it has been shown that NS3 is responsible for the activation of the MAPK/ERK pathway by interacting with the serine/threonine-protein kinase B-Raf. Although beneficial for the virus replication, this phenomenon is likely to contribute to increased expression of pro-inflammatory factors that would cause tissue damage [26].

In addition to its role in inducing apoptosis, it has also been suggested that an oligomer of NS3 possess a viroporin-like activity [29], which could induce plasma membrane permeation, similar to observations on the rotavirus NSP4 protein [30]. Indeed, rotavirus viroporin induces an increase of cytosolic calcium concentration, that is required for the formation of vesicular puncta surrounding the sites of replication. To form this viroporin, NS3 is likely to form oligomers, possibly through a predicted coiled-coil motif located in its N-terminus. However, the presence of NS3 dimers have only been identified in purified NS3 expressed in bacteria [31] and remains to be confirmed in infected eukaryotic cells.

Overall, BTV-induced apoptosis and activation of the pro-inflammatory pathways are the main events leading to BTV lytic release from infected mammalian cells. When infected with BTV in vitro, almost all mammalian cells lines, such as PT cell line (derived from sheep) or BSR cell line (derived from BHK-21 cells from hamster), are lysed after 1 to 4 days post infection, depending on the initial multiplicity of infection. However, several studies on NS3 mutant viruses revealed that when non-lytic release of BTV is inhibited, BTV is still able to spread from cell to cell but is highly attenuated, suggesting that lytic release is not the main mode of BTV egress [17,18,32].

## 3. Budding of a Non-Enveloped Virus at the Plasma Membrane

In the 1960s, observations that purified BTV particles could sometimes be found surrounded by an envelope [33] suggested that this transient envelope was derived from the infected cells. Direct observation of infected cells by electron microscopy later revealed that BTV particles are released by budding at the plasma membrane, where particles acquire an envelope, in contrast with naked particles that are released by membrane extrusion [34]. Analysis of the NS3 protein localisation by immunogold-labelling and electron microscopy revealed that NS3 is present at the plasma membrane surrounding budding virus particles [35]. Budding of BTV structures has also been observed in insect cells. Expression of the BTV inner and outer capsid protein using a baculovirus system, stimulated the release of virus-like particles (VLPs) via budding. This, however, was dependent on the co-expression of the NS3 protein, further implicating NS3 in BTV morphogenesis and release [15].

## 4. The Membrane Protein NS3 is Responsible for Trafficking and Release of the Progeny Virions

The NS3 protein is encoded by segment 10, along with NS3A, an N-terminally truncated isoform of NS3, translation of which initiates from a second start codon at position 14 [36]. To date, no molecular structure of NS3 is available, however it is known to possess two transmembrane domains, connected by a 21 amino acid loop that includes a glycosylation site, and is located in the lumen of the endoplasmic reticulum after protein translation [14]. Although the NS3 amino acid sequence is highly conserved across all BTV serotypes [37,38], certain key amino acids have been linked to variation in BTV virulence [39]. During the infection, NS3 acts as a molecular bridge between the newly assembled BTV particles and cellular factors hijacked to support virus release. The NS3 protein traffics through the endoplasmic reticulum and the Golgi apparatus toward the plasma membrane [18], and completes the BTV replication cycle by facilitating virus egress, particularly via the non-lytic route, as opposed to cell lysis. NS3 possesses two polybasic motifs (PBM1 and PMB2) that are conserved among the *Orbiviruses*; both are located upstream of the first transmembrane domain of NS3 and have recently been characterized, showing that PBM1 acts as a ER retention signal whereas PBM2 is as a membrane export signal [18]. In the case of BTV, the deletion of the polybasic motifs attenuates the virus, such that the majority of released particles are immature cores that are released due to cell lysis, indicating that NS3 is also essential for the maturation of BTV particles, and not only for virus egress. Similar trafficking signals were also found in the FAST protein of the reovirus, or the fusion protein of the Nipah virus [40,41]. Further, although it has been suggested that the VP5 protein possesses an independent membrane export signal [42], the depletion of the PBM domains inhibits VP5 export to the plasma membrane [9]. Recently, we have identified the presence of the NS3 protein in close association with the VIBs in the early hours of the infection [9]. This result suggests that NS3 might trigger ER remodeling to associate with the VIBs, and thereby facilitate the export of core particles, possibly already encapsidated by VP5, from the VIBs to the site of release. While the C-terminal domain of NS3 interacts with both VP5 and the outermost protein VP2, the precise location of the VP5 and NS3 interactions remains to be identified [16,42]. In contrast to its interaction with VP2 and VP5 via its C-terminal domain, the N-terminal domain has a role in hijacking several host factors involved in exocytic and vesicular sorting pathways. Among them, the thirteen first residues of NS3, that are not present in the NS3A isoform, form an amphipathic helix acting as an Annexin II protein-binding motif [16]. The annexin II protein is involved in Ca^++^ dependent exocytosis [43], and is often involved in the maturation and egress of enveloped viruses, such as measles virus or hepatitis C virus [44,45]. In the case of BTV, inhibition of the NS3-Annexin II interaction decreases the release of progeny particles. Moreover, mutant virus with a truncated NS3 lacking the Annexin II interaction domain are highly attenuated, with progeny virions scattered in the cytoplasm of infected cells, and no budding events observed at the plasma membrane [46]. NS3 also harbors two late domain motifs, PSAP and PPRY, which are responsible for binding the TSG101 protein and the NEDD4 ubiquitin ligase respectively [47]. TSG101 is a component of the ESCRT-I complex, regulating the vesicular trafficking process and multivesicular bodies (MVBs) formation [48]. As with Annexin II, many enveloped viruses such as Ebola virus or HIV recruit TSG101 for virus particles budding at the plasma membrane, stressing the significant similarities in egress mechanisms used by the non-enveloped BTV and the enveloped viruses [49]. In the case of BTV, depletion of the Tsg101 binding motif in NS3 inhibits the virus budding at the plasma membrane, but does not interfere with the replication cycle of the virus [17,47]. Depletion of the second late domain motif PPRY also reduces virus shedding, with the majority of particles remaining outside intracellular vesicles rather than inside for the WT BTV particles [50]. Another striking similarity between BTV and enveloped virus maturation is the involvement of the lipid phosphatidylinositol (4,5) bisphosphate (PI(4,5)P2), which is a key effector of virus release [51,52]. It has been reported that PI(4,5)P2 co-localises with NS3 and VP5, and that depletion of this lipid reduced BTV maturation and release [53].

**Figure 2:**
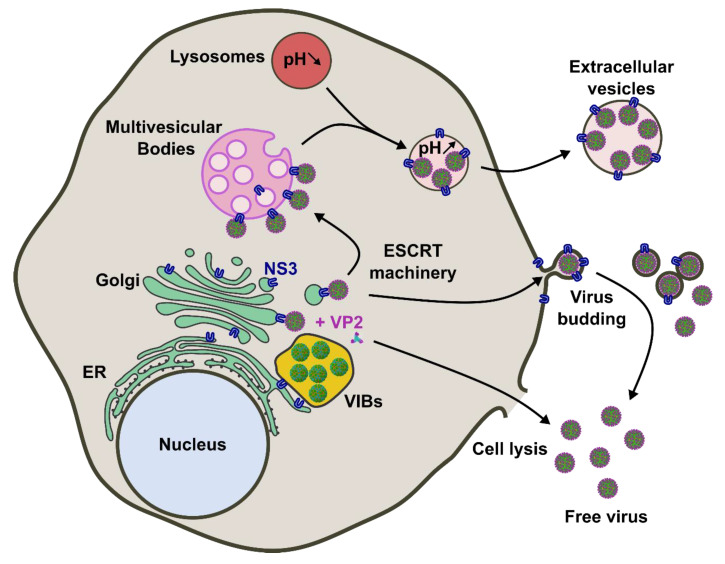
Current model of BTV egress mechanisms from in vitro infected mammalian cells. After the assembly of core particles in VIBs, NS3 (in blue) is responsible for trafficking the BTV particles toward MVBs. Virions are then transferred in secretory lysosomes where the pH is increased from acidic to neutral range, thus preventing virion degradation. Lysosomes are then secreted and BTV is transmitted to another cell as a pool of virions cloaked in EVs. Progeny BTV virions are also exported to the plasma membrane, mediated by the interaction between NS3 and outer capsid proteins of BTV, and between NS3 and the cellular ESCRT machinery. BTV virions are then released by budding at the plasma membrane, afterwards only a fraction of virions keeps a transient envelope. In addition, apoptosis triggered by the infection leads to cell death and the release of free virus particles between 12–24h post-infection. ER: Endoplasmic reticulum, Golgi: Golgi apparatus, VIBs: Viral inclusion bodies, and NS3 is represented in blue.

Overall, the NS3 protein is a key mediator of virus maturation and egress, by interacting with immature cores at the interface of the ER and the VIBs, and by driving complete particles to egress at the plasma membrane (Figure 2). Recently, new evidence suggests that NS3 is involved in the release of BTV particles in extracellular vesicles.

## 5. BTV Release in Extracellular Vesicles

In recent years, a number of reports have shown the non-lytic release of viruses, traditionally regarded as non-enveloped within membrane enclosed carriers [54]. Picornaviruses, for example, have been reported to be released in ER derived EVs containing several virus particles which are then collectively transmitted to another cell [55]. In the case of the rotavirus, another member of the *Reoviridae* family, it was shown that virus particles can be released in EVs putatively derived from the plasma membrane [56], in contrast to other viruses such as the hepatitis B virus, that are released in exosomes derived from the MVBs [57]. Moreover, the type of extracellular vesicles in which these virus particles are released is dependent on the time of infection, as shown for encephalomyocarditis virus [58]. The collective transmission of multiple virions within a single membrane enclosed structure, termed en bloc transmission, is believed to enhance infectivity and modulate the impact on transmission of variation in virion fitness [55,56].

In BTV, particles have been observed both in intracellular and extracellular vesicles. Notably, BTV is known to have a close association with multivesicular bodies (MVBs), via the interaction between NS3 and NEDD4 ubiquitin ligase. Inhibition of this interaction inhibits virus maturation and release [50]. BTV is also known to interact with autophagy pathways within mammalian cells. Specifically, BTV infection of mammalian cells has been demonstrated to induce autophagy, whilst exogeneous induction of autophagy has been shown to promote BTV infection. Conversely inhibition of autophagy inhibits BTV replication, delaying the accumulation of NS3 and the outer capsid protein VP2, thereby reducing virus maturation [59]. Together, these findings demonstrate a clear link between intracellular vesicles and virus maturation and egress.

Recently, we reported that in some cell types, a majority of BTV particles can be released in extracellular vesicles, both in vitro and in vivo [19]. The release of BTV containing EVs was initially confirmed by electron microscope analysis of EVs purified from infected cells culture’s supernatants and blood from BTV infected cattle. During subsequent analysis, these EVs demonstrated increased infectivity compared to the free virus in cell culture, although their importance to pathogenesis in vivo remains to be confirmed. Biochemically, BTV infection triggers an increase of the lysosomal membrane protein LAMP1 at the plasma membrane, and increases the pH of lysosomes, from acidic to neutral range, both of which are indicative of an increase in the exocytosis of secretory lysosomes [60,61].

In addition, inhibition of the MVBs or autophagous lysosomes was shown to decrease the release of infectious EVs. Together these data suggest a model in which nascent BTV particles transit through the MVBs towards the lysosomes. Lysosomes carrying the virus are subsequently trafficked to the cell membrane and released as extracellular vesicles (Figure 2).

## 6. BTV Non-Lytic Release in Insects

Much of the work discussed above has focused on the egress of BTV from mammalian cells. BTV is transmitted to ruminants by biting midges from the *Culicoides* species, and establishes a persistent, non-lethal infection in the vector species. A similar scenario is visible in KC cells, a *Culicoides* derived cell line; BTV replicates to a high titre in these cells but does not induce cytopathic effect. This suggests virion particles are released from insect cells by a non-lytic mechanism [62]. In contrast to mammalian cells, *Culicoides* vector cells respond to BTV infection using different mechanisms involving apoptotic pathway inhibition and RNA interference [63,64]. Here, virus budding is the norm for egress, a situation which is mirrored by the requirement of NS3 for non-lytic release of recombinant VLPs produced in *Spodoptera frugiperda* (caterpillar) insect cells using recombinant baculoviruses. VLPs release could only be achieved by co-expression of NS3/3A [15]. Additionally, NS3/3A is essential for the propagation of BTV in vector species, as knockout of this gene was found to inhibit infection in *Culicoides sonorensis* [65]. As in mammalian cells, budding of BTV from insect cells is dependent on the interaction of NS3 with Annexin II. Of note is that both NS3 and NS3A isoforms are required for efficient virus release from insect cells [16,45]. In contrast to the infection of mammalian cells, the outer capsid of BTV seems less important during the infection of insect cells. It has been reported that infectious subviral particles (ISVP, mature particles treated with trypsin) are more infectious than mature particles in insect cells, both in cell culture and in *Culicoides* vectors [66]. In insects, it was shown that the VP2 protein of BTV particles ingested by *Culicoides* vectors is cleaved by a trypsin-like protease present in the insect saliva [67]. BTV particles treated with insect saliva were more infectious in *Culicoides* derived KC cells and less infectious in mammalian cells than non-treated virus particles. It was also shown that the core protein VP7 can bind KC cells surface [68], suggesting BTV entry into insect cells is independent of the outer capsid. In support of these findings, analysis of *Culicoides* captured in nature revealed that segments 3 and 7, encoding the core proteins VP3 and VP7, were present in 30% of field-collected insects, whereas segment 2, encoding the VP2 protein, was detected in only 16% of the collected insects [69]. Altogether, these results suggest that outer capsid proteins may not be necessary and are downregulated during infection of the *Culicoides* vectors.

## 7. Conclusions

In recent decades, extensive study of the non-structural proteins of BTV, and of the infection of mammalian and insect cells has significantly enhanced our understanding on virus replication processes. Among the key discoveries, the predominantly non-lytic release of BTV, the importance of the NS3 in mediating virus egress, and the relative absence of a viral envelope represent a shift of the egress paradigm, from lytic to non-lytic. However, several challenges remain to further understand how BTV and other arthropod-borne *Orbiviruses* can infect both animals and their insect vectors, with striking variations in the severity of symptoms. Further characterization of the NS3 molecular structure in a lipid environment represents a clear future goal, as this protein is central in host-pathogen interaction. Such structural information would help to understand how NS3 functions as a viroporin while mediating virus maturation and egress. Additionally, although the NS1 protein is known to upregulate viral protein synthesis, NS1 polymer tubules has been hypothesized to play a role in particle trafficking and egress [70]. Thus, the spatio-temporal co-ordination of NS1’s function with that of NS3 needs to be explored further. It is also not clear how NS2 and NS3 communicate to promote virus particle maturation. No doubt the availability of various high-resolution microscopic techniques will be able to address these issues in the near future. Similarly, the role of lipids in BTV infection and replication process offers an interesting subject to be investigated, as it has been observed that in addition to NS3, VP5 possess an independent lipid raft targeting signal, similar to the VP4 and NSP4 proteins of rotavirus [42,71]. Future studies addressing these questions will help to better understand the BTV life cycle in cells, as well as the pathogenicity and ecological cycles among alternating hosts.
